# Hemodynamic Profiles Before and After Surgery in Bicuspid Aortic Valve Disease—A Systematic Review of the Literature

**DOI:** 10.3389/fcvm.2021.629227

**Published:** 2021-03-24

**Authors:** Daniel G. W. Cave, Hannah Panayiotou, Malenka M. Bissell

**Affiliations:** Department of Biomedical Imaging Science, Leeds Institute of Cardiovascular and Metabolic Medicine, University of Leeds, Leeds, United Kingdom

**Keywords:** bicuspid, bicuspid aortic valve, 4DFlow MRI, aortic, aortopathy, aortic valve replacement, transcatheter aortic valve implantation

## Abstract

Bicuspid aortic valve (BAV) disease presents a unique management challenge both pre- and post-operatively. 4D flow MRI offers multiple tools for the assessment of the thoracic aorta in aortic valve disease. In particular, its assessment of flow patterns and wall shear stress have led to new understandings around the mechanisms of aneurysm development in BAV disease. Novel parameters have now been developed that have the potential to predict pathological aortic dilatation and may help to risk stratify BAV patients in future. This systematic review analyses the current 4D flow MRI literature after aortic valve and/or ascending aortic replacement in bicuspid aortic valve disease. 4D flow MRI has also identified distinct challenges posed by this cohort at the time of valve replacement compared to standard management of tri-leaflet disorders, and may help tailor the type and timing of replacement. Eccentric pathological flow patterns seen after bioprosthetic valve implantation, but not with mechanical prostheses, might be an important future consideration in intervention planning. 4D flow MRI also has promising potential in supporting the development of artificial valve prostheses and aortic conduits with more physiological flow patterns.

## Introduction

Bicuspid aortic valve (BAV) is the commonest congenital cardiac condition, affecting ~0.5–1% of the general population (1). It is the leading cause of aortic valve disease under the age of 70 in developed countries and there is an increasingly recognized burden of disease associated. More than 20% of asymptomatic individuals will require surgical intervention in their young adult life (2) and at least 50% patients with BAV will undergo aortic valve replacement at some point in their lifetime (3). Aortopathy, another common comorbidity, occurs in association with BAV in 40–50% of adults (2). The risk of aortic dissection, often a catastrophic event, is ~4%, far higher than the general population, therefore all BAV patients require surveillance (4). The thoracic aorta distal to the sinotubular junction is often not clearly visualized by echocardiography, therefore secondary imaging modalities are frequently required for detailed evaluation. Cardiovascular magnetic resonance imaging (MRI) is the preferred choice to cardiovascular computed tomography as repeated surveillance does not accumulate radiation exposure (5, 6).

Three-dimensional cine (time-resolved) phase contrast MRI with 3D velocity-encoding (4D flow MRI) offers a range of quantitative and qualitative tools to evaluate blood flow through the heart and great vessels over the cardiac cycle. 4D flow MRI has been validated for accuracy and consistency against two-dimensional phase contrast MRI (2D cine PC-MRI) (7) and the techniques have been optimized to now achieve image acquisition in less than 5 min (8). 4D flow MRI provides novel parameters that aid understanding of hemodynamic changes across the aortic valve and thoracic aorta that occur in BAV. 4D flow MRI has also contributed to the knowledge of flow disturbance after aortic valve replacement (AVR), and how this may differ in BAV.

### Common 4D Flow MRI Parameters

In this review we will be concentrating on the 4D flow MRI parameters most frequently researched in BAV disease: helical flow, flow displacement/flow angle, and wall shear stress (WSS). The 4D flow MRI consensus statement presents a full summary of advanced imaging techniques available in vessel 4D flow MRI as well as their potential utility (8).

#### Helical Flow

Laminar flow describes the behavior of a viscous fluid along a pipe. This smooth flow is intrinsically related to the geometry of the vessel and the resistance to the flow (9). Early studies into 4D flow MRI observed the mild helical flow of blood as it travels along the arch of the aorta in normal subjects (10, 11). Helical rotation of blood consistently follows a right-handed (clock-wise) pattern through the ascending aorta (AAo) and aortic arch, and this is preserved throughout life in health (10). Further to this, Kilner et al. discovered an individualized variation in this pattern that could be predicted by aortic arch curvature (11). Computational fluid dynamics (CFD), an alternative method of representing multidirectional flow volumes, has described the same right-handed helical nature of aortic arch blood flow and its crucial relationship to aortic geometry (12).

**Figure 1:**
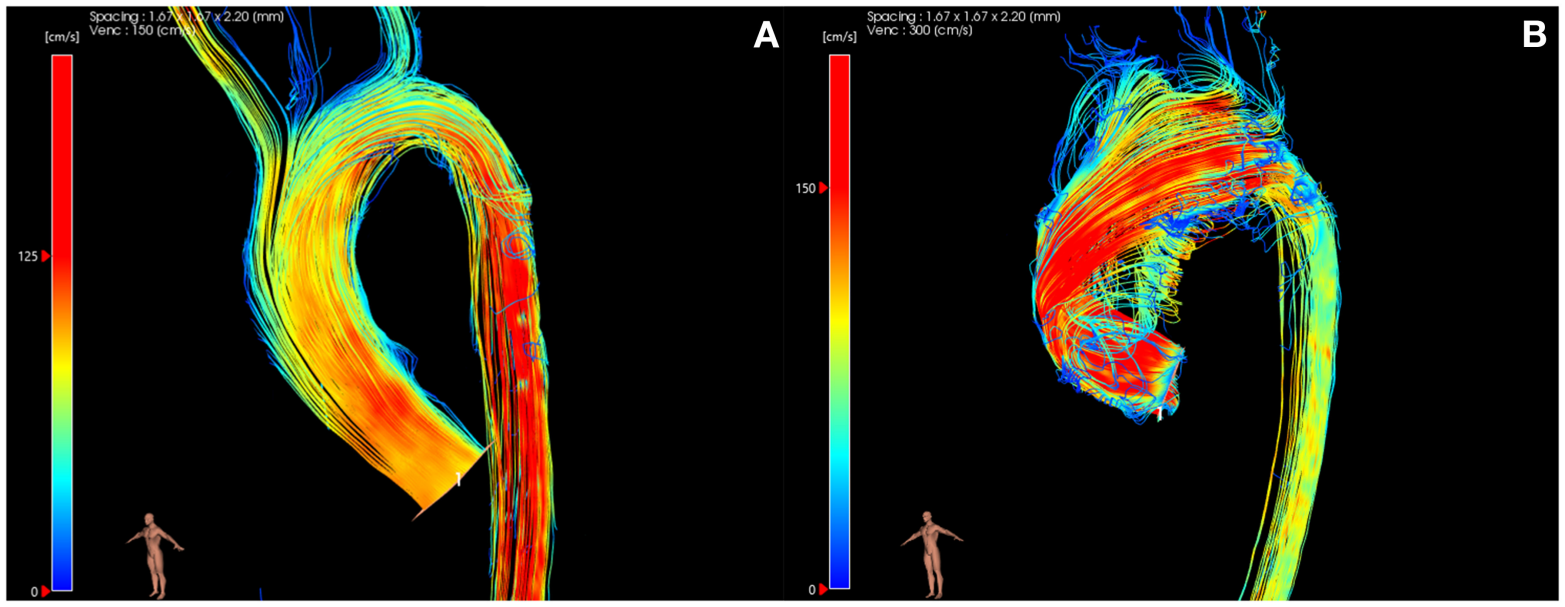
4D flow MRI images showing right-handed helical flow in normal **(A)** and BAV **(B)** subjects.

Initial studies of 4D flow MRI in BAV patients revealed markedly accentuated systolic flow patterns in the ascending aorta, with a more pronounced right-handed helix that persisted into the descending aorta, which is not seen in healthy subjects (Figure 1) (13). 4D flow MRI used in conjunction with 2D analysis planes along the length of the thoracic aorta has allowed quantification of rotational flow (6). A 2013 study of 47 healthy volunteers was used to establish normal limits of helical flow to help differentiate between normal and increased right-handed helical flow providing classification of normal and abnormal aortic hemodynamics beyond subjective image interpretation (6). BAV patients with pathological right-handed helical flow had increased aortic diameters (indexed to body surface area) proportional to the severity of flow disturbance. Conversely, in a smaller group of BAV patients with normal flow patterns, the aortic size was similar to controls (6). This supports the hypothesis that helical flow is an important consideration in the pathogenesis of aortic dilatation in BAV. However, this method alone is not sufficient to determine the causal and temporal relationship of flow pattern and aortopathy.

Preliminary longitudinal studies of 4D flow MRI have examined the association between flow parameters and the rate of aortic growth. A small study of 19 children and young adults with BAV identified a positive correlation between baseline ascending aortic peak velocity and increased ascending aortic diameter growth (z-scores) at follow-up (14). However, deranged patient-specific helical flow patterns remained relatively stable during the study period (14).

#### Flow Displacement and Flow Angle

Another promising parameter for assessing aortic pathology is flow displacement. This is the difference between the central aortic valve plane and the point of maximum velocity-weighted forward flow (15). A similar parameter is the flow angle, the angle between the centerline and the systolic flow jet (16). Flow displacement is higher in BAV than in tricuspid aortic valves (TAV) for healthy and aortic-size matched controls (15). Mahadevia et al. reported a flow displacement, measured at the sinotubular junction (STJ), of 6–8 mm from the anatomical center (17). In a study of pediatric patients with BAV, den Raijer et al. reported that the angle of the systolic aortic flow jet was deviated by 17.5° from the anatomical axis of the aortic valve, compared to 10° in healthy volunteers (18). Furthermore, the flow jet angle was positively correlated with aortic diameters at the sinotubular junction and AAo, suggesting a association with aortic diameters in BAV patients. In addition, both flow jet angle and AAo diameter were significantly associated with elevated levels of matrix metallo-proteinase 2 (MMP-2), a protein implicated in vascular remodeling and aneurysm formation (18–20).

To date flow displacement has demonstrated the greatest predictive ability for aortic growth rate. In a small study of adults with BAV (average follow up 4.3 years), patients with higher flow displacement had a growth rate of 1.2 mm/year compared to 0.3 mm/year for other BAV patients (21). No patients had significant valvular impairment or other risk factors. Peak velocity was also correlated with growth rate (21). A similar study using 2D cine PC-MRI to measure baseline flow displacement found a strong correlation with clinically significant aortic growth over 3 years (22). The authors propose a threshold of 0.2, as above this level rates of growth were four times faster. Displacement was more predictive of aortic growth than baseline aortic diameter or any other flow parameter (22). This finding is supported by the relationship between restricted cusp opening angle, and therefore aortic flow deviation, with higher growth rate (23).

#### Wall Shear Stress

WSS is the frictional drag force exerted by flowing blood on the luminal surface of the vessel (24). It is a computed parameter that has been studied for its important role in the pathogenesis of a variety of conditions, including atherosclerosis and cerebral aneurysms (25–27). Multiple pathways have been identified, revealing endothelial cell morphology, alignment and gene expression alteration, as well as inflammation and dysregulation of the extracellular matrix in response to WSS stimuli (24–27). WSS quantification by 4D flow MRI uses analysis of spatial velocity gradients to calculate WSS as a vector quantity for complete arterial sections (28). Overall WSS can be split into axial (through-plane) and circumferential (in-plane) WSS.

BAV patients exhibit disturbed WSS in the AAo and aortic arch (6, 29, 30). In BAV patients with right-handed helical flow, peak systolic axial WSS is significantly raised compared to controls (6, 29). WSS is asymmetrical and eccentric in the AAo, with peak values on the right-anterior wall corresponding to flow jet direction (29). Circumferentially-averaged WSS (WSS_circavg_) averages the WSS in a given 2D plane within the vessel and is persistently elevated in right-handed flow BAV patients, even at increasing aortic diameters (6, 30). This is in contrast to healthy volunteers, which demonstrate a decrease in WSS_circavg_ as aortic diameter increases (6), resulting from the same blood volume traveling through a larger vessel and therefore exerting less friction against the vessel wall.

WSS distribution in the aorta in BAV has been modeled using CFD, and applied *in vitro* in a porcine aorta (31). On subsequent histological analysis, aortic tissue exposed to BAV WSS showed higher expression of MMP-2 and MMP-9 that was not seen in controls (31). McNally et al.'s *in vitro* experiments of non-dilated BAV aortas have demonstrated substantial flow and WSS disturbance that predicted the regions prone to dilatation (32). A landmark study by Guzzardi et al. confirmed these findings in humans and showed that the histopathological changes seen in BAV aortopathy were only present in areas of high WSS, but not in those with normal or low WSS (33). These important findings indicate that WSS plays a vital role in initiating and driving aortic dilatation.

#### Aortic Dilation and Flow

To further clarify the impact of the aneurysmal aorta on flow, it is important to examine other causes of aortic dilatation. In a study of TAV patients with thoracic aortopathy (diameter >40 mm) from various etiologies, accentuated right-handed helical flow patterns were observed in nearly all patients (34). In patients with Marfan syndrome, normal flow patterns were seen in the AAo and arch, with local helix formation in the descending aorta (35). However, WSS was significantly lower than in healthy subjects with tricuspid valves (17). A further study by Bürk et al. found reduced WSS throughout the AAo and arch compared to age-matched controls, despite helical flow patterns being seen in aneurysm patients (36). This is in contrast to BAV, where WSS remains stable or increases as aortic size increases. Indeed, the areas of highest WSS correspond with the sites of aneurysm formation (32).

### Aortic Surgery

Aortic diameter is linked to prognosis. Fifteen-year freedom from complications is 86% in patients with ascending aortic diameter less than 40 mm, but falls to 43% for diameter greater than 45 mm (37). Therefore, a key clinical priority is the identification and close monitoring of groups at risk of aortic growth. The presence of a bicuspid valve increases growth rate, with faster growth potentially linked to the aneurysm morphology (38). However, BAV patients still represent a heterogenous group of patients, some remaining asymptomatic into their old age, presenting a challenge for surgical (39).

The 25 year rate of aortic surgery in BAV is 25% (40). AVR is the commonest procedure and may be performed with or without aortic root replacement. Patients with BAV undergo AVR at a younger age than those with degenerative valve disease, and aortic stenosis is the most prevalent indication for valve intervention (41). The rate of proximal (Type A) aortic dissection post-AVR is reported to be 4–14%, though there is significant variation in the published literature (42, 43). Aortic rupture or re-dilatation requiring further surgery also occur (42). AVR has been associated with late aortic complications, but the mechanisms underlying this are not clear (41). There remains uncertainty amongst cardiac surgeons about whether prophylactic aortic replacement should be used to combat these risks in BAV patients (44). In this systematic review we conducted a comprehensive review of the 4D flow MRI literature in AVR and ascending aortic replacement.

## Methods

### Eligibility Criteria

Studies which examined the use of 4D flow MRI for assessment of hemodynamic changes after Aortic Valve Replacement in Bicuspid Aortic Valve disease were included. Inclusion was limited to peer-reviewed literature, original reports, and human participants. Studies not published in English and solely *in vitro* experiments were excluded.

### Search Strategy

A search of Medline and EMBASE was conducted for relevant literature. Search terms were identified, and searches were conducted using the PICO format: *bicuspid aortic valve*; *aortic valve replacement, aortic root replacement, aneurysm repair, ross procedure, TAVI*; *4D flow MRI, 4D flow CMR*. A search of additional sources included PubMed and reference lists of included studies. The search was carried out in February 2021.

### Study Selection

**Figure 2:**
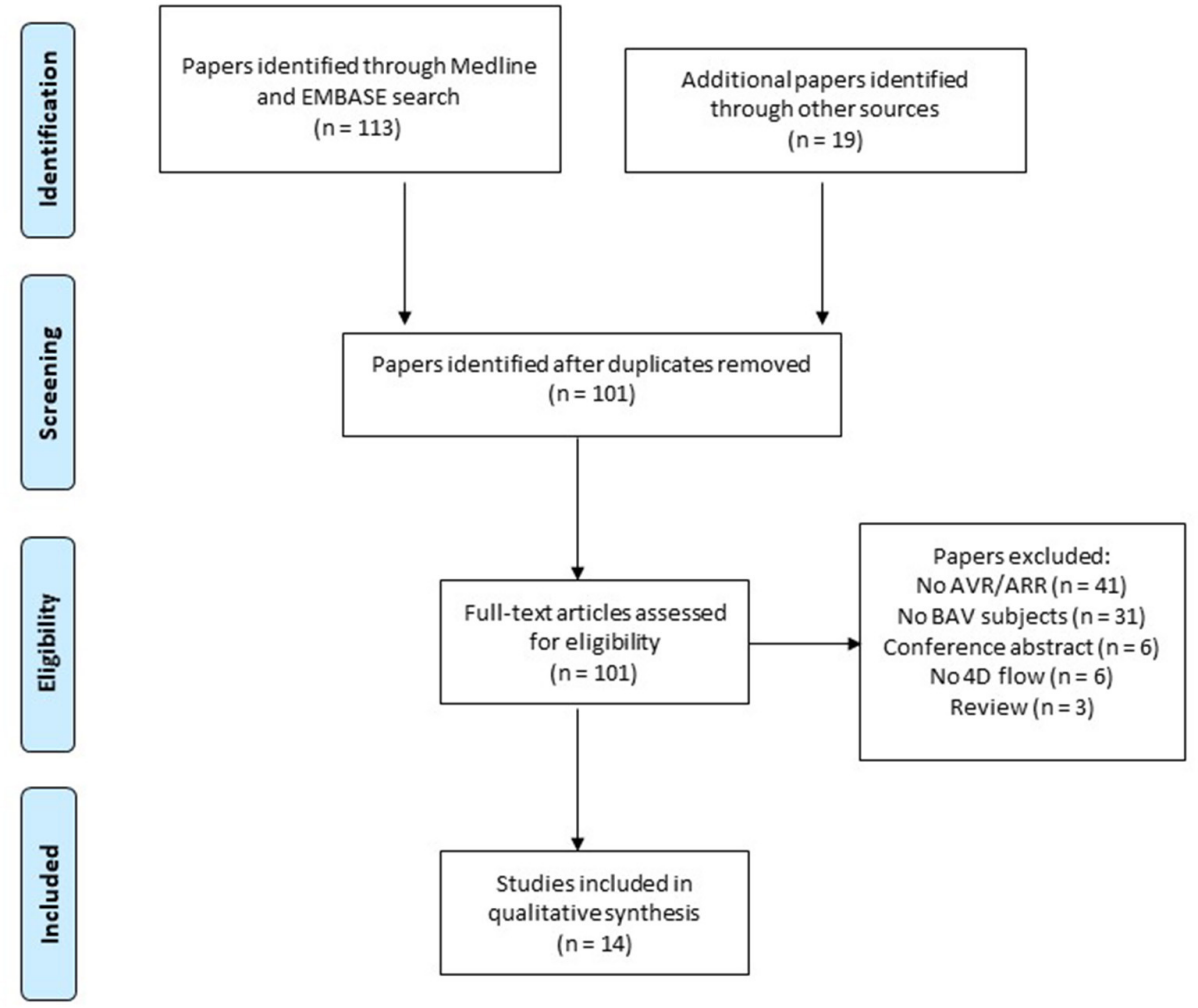
PRISMA flow diagram.

Studies identified by the database search were assessed by two independent reviewers. The review of the proposed papers was conducted by DC (2 years experience as a doctor) and HP (4 years experience as a doctor). For disagreements, manuscripts were reviewed and decied upon by a third reviewer MB (11 years of 4D flow MRI research experience). PRISMA guidelines were used to identify relevant studies. Duplicate studies, review articles, conference abstracts, and those not meeting the search criteria were excluded. Papers using 4D flow MRI in Aortic Valve or Root Replacement were reviewed individually, and included if patients with bicuspid valves participated. Results of the study selection are shown in the PRISMA flow diagram (Figure 2).

## Results

After a systematic search using multiple databases, we identified 101 studies for detailed consideration. After review of all full-text articles, a further 87 were excluded for the following reasons: did not mention aortic valve or root replacement (45); did not include BAV patients (31); did not use 4D flow MRI (6); conference abstracts (6); reviews (3). The remaining 14 studies were included for review of methodology and key results, with particular focus on Bicuspid Aortic Valve disease. The included papers are summarized in Table 1 below.

**Table 1:** Summary table of included studies.

**References**	**N**	**Study population**	**Surgical procedure**		**Valve type**	**4D flow Parameters**				**Main findings**
			**AVR**	**ARR**		**WSS**	**PV**	**FD/FA**	**Flow pattern**	
Kamada et al. (46)	10	BAV & TAV Aortic stenosis	+		Mechanical				+	mAVR reduced Flow Angle and helicity density in the ascending aorta.
Bollache et al. (47)	53	BAV & TAV aortopathy	+	+	Mechanical, bioprosthetic, valve-sparing	+				Peak WSS reduced after mAVR but increased in ARR distal to the graft.
Bissell et al. (48)	90	BAV	+		Mechanical, bioprosthetic, Ross	+	+	+	+	Abnormal flow pattern in all after bAVR but only 27% after mAVR.
Farag et al. (49)	35	BAV	+		Mechanical (bileaflet)	+	+			Mean WSS after mAVR is greater than healthy control but similar to native BAV.
Von Knobelsdorff-Brenkenhoff et al. (50)	47	BAV & TAV	+		Mechanical, bioprosthetic, Ross	+			+	AVR resulted in asymmetrical flow and WSS compared to Ross and control.
Keller et al. (51)	20	BAV & TAV aortopathy	+	+	Mechanical				+	Reduction in abnormal flow patterns after mechanical ARR.
Collins et al. (52)	37	BAV & TAV aortopathy	+	+	Bioprosthetic, valve-sparing		+		+	Lower PV and improved flow pattern with valve-sparing ARR.
Condemi et al. (53)	1	BAV aortopathy		+	Valve-sparing	+			+	Reduced helicity but marked increase in velocity after ARR.
Stephens et al. (54)	24	BAV aortopathy		+	Valve-sparing	+			+	After ARR, Type 1 (R-L) had higher WSS compared to Type 0 BAV.
Allen et al. (55)	1	BAV aortopathy		+	Valve-sparing		+		+	Greatly increased PV after valve-sparing ARR.
Semaan et al. (56)	33	BAV & TAV aortopathy		+	Valve-sparing		+		+	Higher PV after ARR in bicuspid patients. Improved flow pattern after ARR in all.
Oechtering et al. (57)	36	BAV & TAV aortopathy		+	Valve-sparing				+	Altered flow patterns after ARR due to altered aortic geometry.
Gaudino et al. (58)	30	BAV & TAV aortopathy		+	Valve-sparing	+			+	Neosinus reconstruction resulted in physiologic vortices and reduced WSS.
Oechtering et al. (59)	30	BAV & TAV aortopathy		+	Valve-sparing		+		+	Similar sinus vortex pattern to healthy controls with valve-sparing sinus prosthesis.

*AVR, aortic valve replacement; ARR, aortic root replacement; WSS, wall shear stress; PV, peak velocity; FD, flow displacement; FA, flow angle; BAV, bicuspid aortic valve; TAV, tricuspid aortic valve; mAVR, mechanical aortic valve replacement; bAVR, bioprosthetic aortic valve replacement*.

The 14 selected papers studied a total of 447 participants (range 1–90). Five studies examined 4D flow MRI changes after isolated AVR, and of these two used bioprosthetic valves, two used pulmonary homografts (Ross procedure), and all five included mechanical valve types (46–50). The most commonly reported quantitative flow parameter was aortic WSS (4 out of 5 studies). No studies of TAVI were found that included BAV patients. Ten studies provided 4D flow data on ARR for patients with BAV aortopathy (47, 51–59). The majority (nine) of these studies used a valve-sparing technique (47, 52–59), with two using a mechanical prosthesis (47, 51) and two comparing valve-sparing and valved ARR (47, 52). All ARR studies reported data on the flow pattern or helicity in the aortic arch, with four also reporting WSS, and four reporting PV values. Three studies of isolated AVR identified by the initial search criteria did not provide characteristics of the native valve type of included patients (60–62). These were therefore not included in the summary table, however their results were deemed to be important in understanding post-operative hemodynamic changes, and have been reviewed below.

## Discussion

### Isolated Aortic Valve Replacement

Aortic growth in BAV is accelerated compared to TAV pre-operatively. After AVR, aortic growth continues but is not different between these groups suggesting the AVR influences risk of late complications (63).

**Figure 3:**
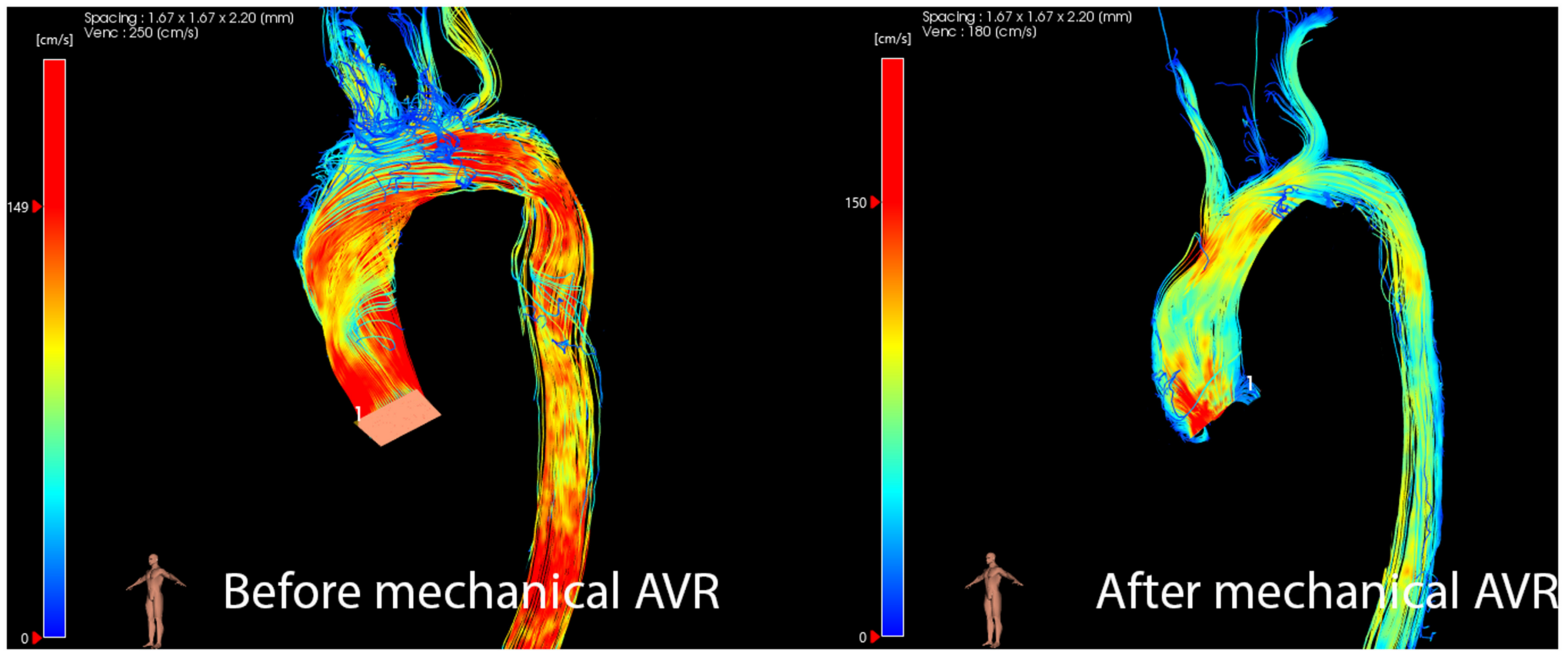
Flow profile normalizes after Mechanical AVR.

Flow characteristics undergo significant change after AVR. In one 4D flow study of patients with aortic stenosis before mechanical AVR and shortly afterwards, flow volume increased by 30% and flow angle in the ascending aorta reduced from 39° to 25°(46). The post-operative flow pattern in the AAo and arch showed a marked reduction in helicity, and streamlines appeared similar to normal volunteers. Furthermore, regional WSS reduced after AVR (47). In a study of 30 patients with BAV undergoing AVR, 73% of patients with mechanical AVR had normal flow patterns post-operatively, with reduced rotational WSS compared to unrepaired BAV controls (48). Flow angle also reduced, and flow displacement after mechanical AVR was similar to healthy subjects (Figure 3) (48). In patients receiving bileaflet mechanical AVR, mean and peak WSS were even lower than healthy volunteers (49). This suggests that these patients might be at lower risk of further aortic remodeling and subsequent dilation.

**Figure 4:**
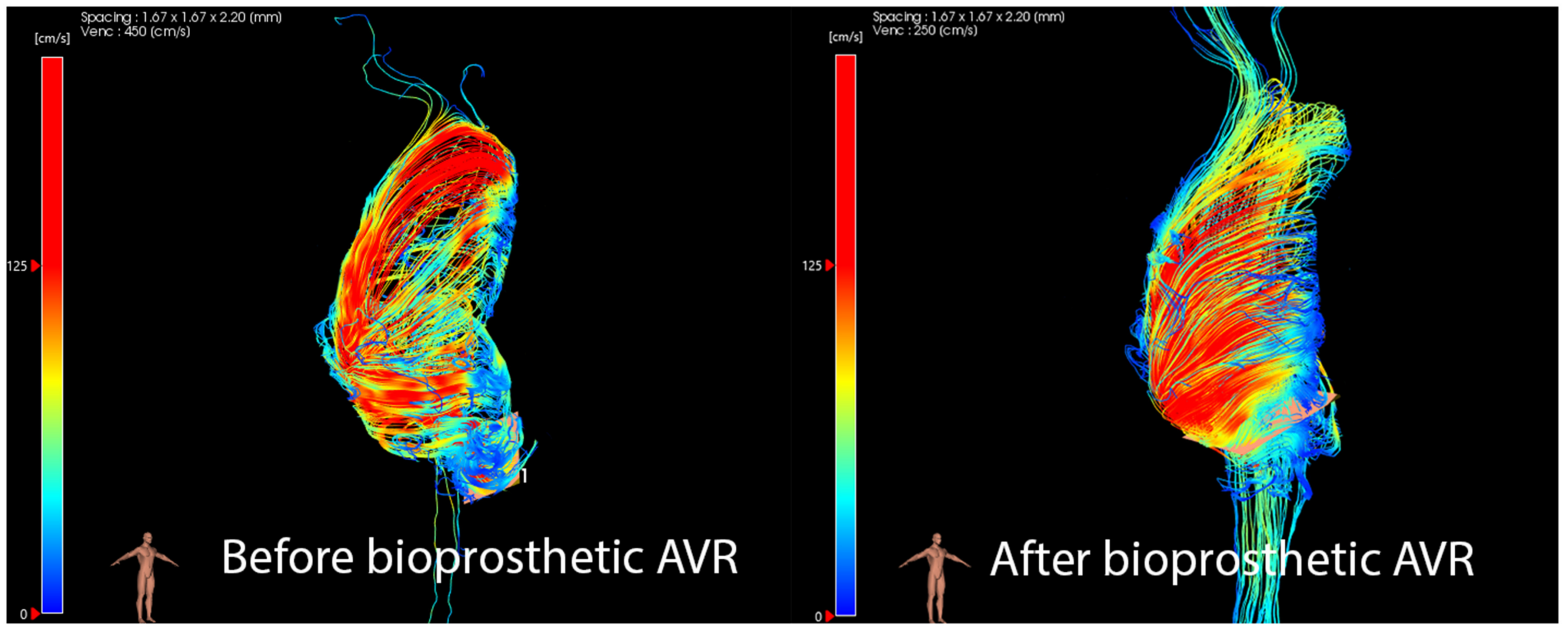
Marked helical flow profile remains after bioprosthetic AVR.

In contrast, patients who receive bioprosthetic AVR continue to exhibit highly abnormal flow patterns, with similar degrees of right-handed flow to unrepaired BAV controls (Figure 4) (48). When measured before and after surgery, rotational WSS and flow displacement did not change, and remained significantly higher than normal (48). In the same study, mechanical AVR patients had dramatically reduced WSS compared to pre-operative levels. However, in another study of 4 patients after bioprosthetic AVR, peak aortic WSS decreased in all patients compared to pre-operative imaging, but only to the level of unrepaired BAV controls. Both stented and stentless bioprostheses cause abnormal helicity and asymmetrical distribution of peak WSS, significantly higher than controls and mechanical AVR patients (50, 61). The flow eccentricity and helicity was slightly reduced in stentless valves, possibly due to the larger functional valve orifice and closer resemblance to normal TAV anatomy (50). Despite these drawbacks, bioprosthetic valves have many advantages including the avoidance of anticoagulation. Utilizing 4D flow MRI to design more physiological valve geometry may improve the longevity of bioprosthetic valves and minimize complications.

The Ross procedure offers a third option for patients needing AVR, involving autografting of the patient's native pulmonary valve to the aortic position. A new pulmonary valve is then usually reconstructed from cadaveric tissue (64). The procedure is often conducted in childhood, as native tissue will grow with the child, and is often used in BAV patients. In the study by Bissell et al., ascending aortic rotational flow values after Ross procedure were similar to mechanical AVR and healthy subjects, and the majority had normal flow patterns (48). Systolic flow angles and flow displacement were also near-normal (48). Conaglen et al. reported that Ross patients had the lowest helicity of all valve types, and even had lower peak WSS than controls at the STJ (65). The Ross procedure therefore returns flow parameters to normal, likely explaining the favorable long-term outcomes with no significant ascending aortopathy, albeit often dilated sinuses (65). It is however technically complex to perform, and may require reintervention of both the right and left outflow tracts in later life (66).

Transcatheter aortic valve implantation (TAVI) is an alternative to AVR in patients with severe aortic stenosis, that avoids the need for sternotomy and cardiopulmonary bypass. To date few studies have examined the hemodynamic effects after TAVI, and none have included BAV patients. One report found TAVI results in increased flow eccentricity and flow displacement in the AAo compared to surgical AVR and controls (62). Others have shown reduced helical flow with TAVI (Edwards Sapien XT) compared to bioprosthetic AVR, but still abnormal compared to healthy controls (60). Patients with BAV undergoing TAVI are more likely to suffer device failure, paravalvular leak and conversion to surgery in one meta-analysis (67), complicating the decision making process.

### Aortic Root Replacement

Prophylactic aortic root replacement is often considered at the time of AVR in patients with aortopathy to mitigate the risk of future aortic dissection. The Modified Bentall procedure is the standard technique, consisting of a synthetic ascending aorta and root graft with a bioprosthetic or mechanical valve (68). A large study has demonstrated the low rate of long-term aortic complications with this technique (69). Previous guidelines established aortic size thresholds for surgical intervention, however these are predominantly based on connective tissue disorders such as Marfan syndrome (70). Clinical data suggest aortic growth and late complication rates differ in BAV compared to intrinsic aortopathies (71). Additionally, so far there is no evidence for accelerated aortic growth rate after AVR compared to prophylactic aortic root replacement (72).

Using 4D flow MRI, hemodynamic effects of the synthetic aortic root graft can be examined in more detail. On blinded visual assessment of helical flow patterns, pre and postoperatively, aortic root replacement with a mechanical (On-X) valve returns flow distortion to normal (51). Peak transvalvular pressure gradients are similar when measured with 4D flow MRI and Doppler echocardiography, and show a decrease after mechanical ARR (51). However, helical flow remains highly abnormal after ARR with a bioprosthetic valve (52). Peak velocities also remain elevated compared to controls and persist into the aortic arch and descending aorta (52). Abnormally high WSS develops in the aorta immediately distal to the graft when assessed before and after ARR with hemi-arch replacement in one study (47); this raises concerns for further remodeling in this region. A possible explanation for this is the reduced compliance of the aortic graft material resulting in increased flow velocities (73). The stiffer material results in loss of the Windkessel effect of large elastic arteries, whereby hydraulic energy is stored in distensible vessels during systole and discharged during diastole, therefore energy loss occurs after ARR (73, 74). This poses the question whether prophylactic ARR just defers the ongoing hemodynamically driven aortic dilation further downstream.

### Valve-Sparing Aortic Root Replacement

For patients with aortopathy and adequate valve function, valve-sparing aortic root replacement is another surgical option for the prevention of aortic complications. This technique is gaining popularity, particularly in younger patients where retaining the native valve is desirable. However, the procedure, developed for tricuspid aortopathies, requires consideration of individual valve geometry if used in BAV. Several reports have examined the flow characteristics after valve-sparing thoracic aortic aneurysm repair in BAV patients (53–55). Similar to valved conduits, valve-sparing aortic root replacement results in higher flow velocities throughout the thoracic aorta (52, 56). Flow velocity increases after surgical repair resulting in higher WSS in the graft and distal aortic regions (55). Two studies have directly compared valve-sparing and valved aortic root replacement, and have demonstrated significantly higher peak velocity and WSS with the valve-sparing technique, particularly in regions distal to the graft (47, 52).

Flow velocity is significantly higher and more eccentric when the reimplanted native valve is bicuspid compared to tricuspid (56). Flow distortion may be exaggerated further by BAV cusp morphology. Stephens et al. evaluated 19 BAV patients after valve-sparing aortic root replacement and found that left-right coronary cusp fusion BAV had elevated and asymmetric WSS throughout the AAo (54). In contrast “purely bicuspid” BAV patients with a single midline commissure had significantly lower WSS similar to healthy TAV controls (54). This may influence long-term risk of distal complications, although longitudinal studies are needed.

**Figure 5:**
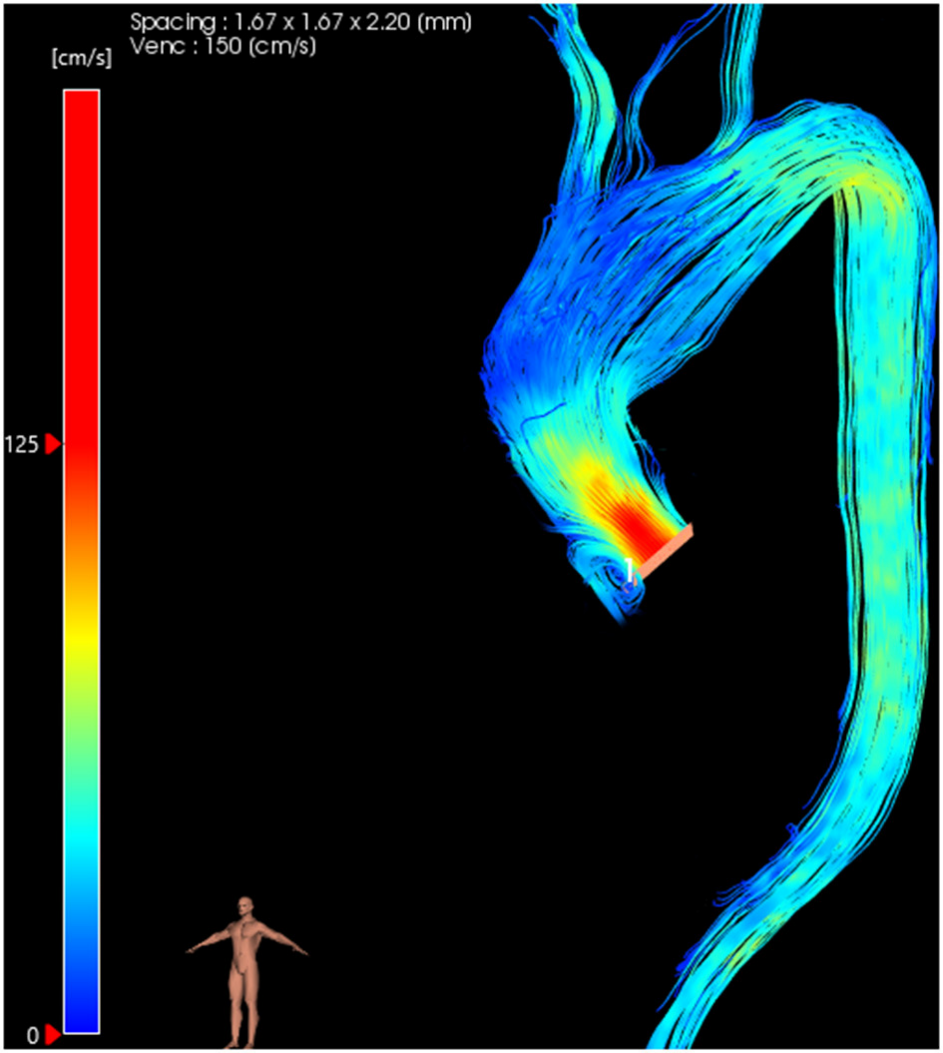
Vortical flow after aortic root replacement.

Aortic reconstruction may also disturb normal aortic root physiology, as assessed by 4D flow MRI. Systolic flow vortices in the Sinus of Valsalva (SOV) occur after peak systole and persist until diastole and are thought to have an important role in valve closure and coronary perfusion (Figure 5) (75). Valve-sparing aortic root replacement, by either the David or Yacoub technique, involves excision of the native Sinus of Valsalva (68). This loss of sinus architecture abolishes vortical flow and may have longer term consequences. New techniques to reconstruct neosinuses during valve-sparing aortic root replacement are effective at restoring vortical supravalvular flow and reducing WSS in the ascending aorta (58, 59, 76).

All valve-sparing aortic root replacement recipients display altered aortic geometry compared to the round shape of healthy controls. This is most often due to kinking of the graft or the distal anastomosis and is observed in around 90% of valve-sparing aortic root replacement patients. Altered geometry is associated with the development of deviated flow patterns (57, 59). In the only longitudinal study of 4D flow MRI after valve-sparing aortic root replacement, 12 patients with Marfan syndrome were followed for an average of 8 years post-operatively (77). One patient developed a Stanford Type B aortic dissection, after demonstrating highly abnormal helical flow patterns and higher normalized flow displacement compared to the other patients (4.5 vs. 1.8%). WSS on the right anterior wall of the ascending aorta was also higher than others in this case (78). Another report found an association with abnormal flow and aneurysm formation after surgery for aortic coarctation (77). To date no longitudinal 4D flow MRI data on BAV patents after valve-sparing aortic root replacement.

### Limitations of 4D Flow MRI

The acquisition of 4D flow MRI data requires additional sequences to be added during the MRI scan which are not always readily available to the clinician. There is the potential for variation in data depending on the equipment (1.5 vs. 3.0T MRI) and sequence used. Further variation can be introduced with the use of different post-processing platforms. Gadolinium-based contrast may sometimes be required during clinical protocols, however this is unlikely to negatively influence 4D flow acquisition and may even enhance flow visualization (79).

While 4D flow MRI has been validated in numerous studies, it is important for individual sequence set up to undergo validation against the local gold standards. Doppler echocardiography is clinically established for measurement of peak velocity and is considered a surrogate gold standard. It is important to also acknowledge that the typically low temporal resolution of 4D flow MRI (40–50 ms) could fail to capture the peak jet, and therefore some underestimation is likely. 2D cine PC-MRI is the gold standard for total forward flow comparison, which is less affected by lower temporal resolution.

## Conclusion

BAV disease presents specific management considerations at the time of surgery and thereafter. 4D flow MRI presents a promising clinical tool to identify BAV patients who may be at risk of developing a more severe aortopathy phenotype, with progressive aneurysm formation and risk of dissection. However, further large prospective studies are needed to confirm this prognostic value for patients and clinicians. 4D flow MRI also shows promising value in supporting the development of artificial valve prostheses and aortic conduits with more physiological flow patterns. The inferior performance of bioprosthetic valves compared to mechanical prostheses in 4D flow studies may explain late post-operative complications and offers opportunities for improved valve design. Valve morphology and the consequent flow patterns are crucial when considering valve-sparing aortic root replacement, and further longitudinal data is needed on the outcomes for patients with bicuspid valves. 4D flow MRI is now fast enough to allow wide spread clinical integration leading to easier facilitation of large multi-center trials.

## Author Contributions

DC and MB contributed to the inception, literature review, and writing of the manuscript. HP was the second reviewer and contributed to the manuscript. All authors contributed to the article and approved the submitted version.

## Conflict of Interest

The authors declare that the research was conducted in the absence of any commercial or financial relationships that could be construed as a potential conflict of interest.
